# A Discovery of a City of Cliff Dwellers

**Published:** 1882-05

**Authors:** 


### POPULAR SCIENCE DEPARTMENT.

### SIR CHARLES LYELL'S ANCESTRY.

Whence did he come ? What conditions went to beget him ? From what stocks were his qualities derived, and why ? These are the questions that must henceforth always be first asked when we have to deal with the life of any great man. For we have now learned that a great man is no unaccountable accident, no chance result of a toss-up on the part of Nature, but simply the highest outcome and final efflorescence of many long ancestral lines, converging at last toward a single happy combination. Whatever he possesses he has derived in the main from his ancestors, though he may possibly add some few elements himself by functional use ; and it is not, perhaps, too much to say that the most richly endowed natures must necessarily derive many of their separate endowments from very different preceding strains. In Lyell's case the ancestral facts are clear and simple. His father was a Lowland Scotch laird, a man of cultivation and refinement, with tastes wide enough to embrace both literature and science. He was a botanist of some distinction, of whose researches into the cryptogams Humboldt himself spoke with favor ; and later in life he became an enthusiastic Dante scholar, collecting every known edition, and publishing numerous translations from the Florentine poet. Thus the father already foreshadowed the special combination of tastes to be found in the son. His mother came from a good Yorkshire family—the Smiths of Maker Hall, in Swaledale—and we can well believe Mrs. Lyell's statement that she was a woman of sound sense, for all her children seem to have inherited more than their father's share of intellect and vigor. Charles was the eldest of ten, having two brothers and seven sisters. All were able, but he was the ablest. The first-born of a wealthy and cultivated family, with ample means and ample leisure, endowed by nature with literary and scientific potentialities, brought up in the stimulating atmosphere of his own home, of Oxford, and of the London literary world, surrounded from his childhood upward by men of science and men of letters, it would have been strange if Charles Lyell had not turned out exactly such a man as we all know him to have been. He was predestined for his work by the inevitable forces of his own constitution and the environing society, and he was admirably fitted beforehand for the work he had to do.—*From "Sir Charles Lyell," by* Grant Allen, *in Popular Science Monthly for March.*

### SOME GROUND BEETLES.

We lived in Fresno County, Cal., two years, in the north-eastern part, and in the foot-hills of the Sierra Nevada. It is hot and dry there ; no trees and many rocks where we were ; the thermometer ranging from 96° to 108° for about three months. In June and July, when hottest and driest, the " overflow bugs" filled the air between sunset and dark. You could not with safety open your mouth. They would light all over your clothes ; they filled the house ; they swarmed on the table, in the milk, sugar, flour, bread, and everywhere there was a crevice to get through. Take a garment from the wall, and you could shake out a cupful. It was a veritable plague. In a shed where the boards had shrunk and the cracks been battened, the spaces between the shrunken boards were packed full. They were flying for about two weeks, and then they disappeared mostly or they did not fly much, but were hidden under papers, clothing, and every available place. In November, before the rains, they spread around, but not to fly ; make a light in the night, and you would see the floor covered ; lift up a rug, and the floor under would be black, and they would go scattering away for some other hiding-place. I had occasion to take up a floor board after they had apparently disappeared, except stragglers. The house was upon underpinning two feet or more from the ground. When the board was raised there were the " overflow bugs" piled up against a piece of underpinning, making such a pile as a half bushel of grain would make. They were all through the foot-hills the same, and much the same in Los Angeles, about Norfolk, but they did not fly much in the latter place. In Los Angeles they seemed to be worse before the " Santa Annas," a hot wind from the desert filling the air with sand ; and though the chickens were ever so hungry for insects, they would not eat the " overflow bugs."

In the night you put up your hand to brush one from your face, and then you get up for soap and water to cleanse your hand. In the morning, if you put on garments without shaking, you get them quickly off and shake them.—*Nature.*

### A DISCOVERY OF A CITY OF CLIFF DWELLERS.

Mr. J. J. Stephenson, the conductor of an exploring expedition to New Mexico and Arizona last fall, communicated to the New York *Tribune* a very interesting account of a discovery of an ancient cliff city sixty miles long.

Mr. Stephenson examined this deserted city during several days, visiting portions distant forty-five miles from each other, and discovering with his glass that the excavations extended fifteen or twenty miles further on. By far the greater number are inaccessible, but many of the old paths, worn many inches deep by the feet of the ancients who dwelt there, are intact, and by them the explorer mounted to the old dwellings. There was a marked similarity in the form and construction of these excavations. There was only one aperture, which served for door, window, and chimney. The single room had an oval roof, which bore the grooves made by the flinty adzes or axes of the excavators. The method of digging or carving out these caves was disclosed by the form and direction of the grooves, which were usually parallel to each other, and several inches apart, while between, as shown by the rough surface of the stone, the remaining substance had been broken off. There were fireplaces at the rear, but no place of exit for the smoke, except the single aperture in front. Many of the dwellings had side or rear excavations of small size, within some of which corn-cobs and beans were found, evidently left by chance inhabitants of a later period. Near the roof of many of the caves there were mortices, projecting from which in some instances there were discovered the decayed ends of wooden sleepers. These were of a kind of wood not recognizable as a present growth of the locality, and unknown to the explorers. Specimens were brought away to be examined and classified by naturalists. In the sides of some dwellings there were found small recesses, evidently used as cupboards for the household utensils of the family. The substance of the cliff was tufa, a volcanic ash quite soft and easily worked by the rude implements of the old builders.

Upon the top of the *mesa*, or table-land, above these caves there were found large circular structures, now in ruins, but with walls to the height of ten or twelve feet still standing. They were evidently places of worship. They were built of square stones of nearly uniform size, about twenty inches in length by six inches in width and four in thickness, cut from the cliff. Measurements were made of two of these structures, one of which was 100 and the other 200 feet in diameter, and might have held from 1000 to 2000 people. The inference that these were places of worship is drawn from the fact that the Pueblos of the present day, who are fire and sun worshippers, have similar temples. No remains of altars were found, which fact is doubtless to be explained by the exposed situation and the soft materials probably used in the construction of such furniture. The southern end of this cave city, which seemed to have been the most densely populated, presented many evidences of art and industry. There were found many animal forms carved out of stone. In one place there were two life-size mountain lions, animals which are still peculiar to that region. There are also to be seen many smaller animal forms, so much worn away that it cannot be determined what they were designed to represent. Upon standing walls in this neighborhood are many hieroglyphics, which from their resemblance to the picture writing of the living Pueblos, may, Mr. Stephenson thinks, be partially, if not entirely deciphered. The great age of this city is proved by the vast accumulation of *débris* from the upper portion of the cliff which covers its base. In places where mountain brooks have cut their way through, the existence of one and sometimes two rows of cave dwellings below the surface of the *débris* is disclosed.—*Phrenological Journal.*

